# A prospective evaluation of the effect of transanal minimally invasive surgery (TAMIS) on low anterior resection syndrome

**DOI:** 10.1007/s00464-023-10004-1

**Published:** 2023-03-24

**Authors:** William P. Duggan, Diarmuid D. Sugrue, Naomi Shannon, Brenda Murphy, John P. Burke

**Affiliations:** 1 Department of Colorectal Surgery, Beaumont Hospital, Dublin 9, Ireland; 2 Department of Physiology and Medical Physics, Royal College of Surgeons in Ireland, Dublin, Ireland

**Keywords:** TAMIS, Low anterior resection syndrome, Quality of life

## Abstract

**Purpose:**

Transanal minimally invasive surgery (TAMIS) is a surgical alternative to transanal endoscopic microsurgery (TEM), transanal excision and proctectomy in the management of benign rectal polyps and early rectal cancers. Low anterior resection syndrome (LARS) describes the constellation of symptoms which result from and are common after distal colorectal resection. Symptoms include incontinence, frequency, urgency and evacuatory dysfunction. The aim of the current study was to prospectively evaluate pre- and post-operative LARS in patients who undergo TAMIS.

**Methods:**

We conducted a prospective analysis of a consecutive series of patients who underwent TAMIS at our institution between January 2021 and February 2022. A LARS questionnaire was undertaken preoperatively, at 1 month and at 6 months post-operatively.

**Results:**

Twenty patients were recruited to this pilot study. The mean age was 63 ± 12 years, 11 of the patients were male, mean pre-operative BMI was 29 ± 6 kg/m^2^, and 30% (*n* = 6) of patients underwent TAMIS for an invasive rectal cancer, with all patients receiving an R0 resection. Mean distance from the anal verge was 5.7 ± 3.2 cm, and mean lesion diameter was 46 ± 20.5 mm. A statistically significant interval reduction was observed between preoperative (20.3 ± 12.9) and 6-month post-operative (12.6 ± 9.7) LARS scores (*p* = 0.02) and also between 1-month (18.2 ± 10.6) and 6-month post-operative scores (*p* = 0.01).

**Conclusions:**

We noted a high prevalence of LARS across our cohort preoperatively, and this had improved significantly at 6-month review post-TAMIS. This study reaffirms the safety and efficacy of TAMIS for the treatment of early rectal neoplasia.

Low anterior resection with total mesorectal excision (TME) remains the gold standard treatment of rectal cancer [1]. Unfortunately, low anterior resection is often associated with a troublesome myriad of symptoms which significantly impact quality of life (QOL), including faecal incontinence, increased frequency of bowel movements, urgency and tenesmus. Together, these symptoms are known as ‘low anterior resection syndrome’ (LARS). LARS is reported in up to 90% of patients following low anterior resection and has been found to correlate with a significant decrease in QOL [2, 3].

The widespread implementation of national screening programmes has led to a significant increase in rates of detection of early colonic and rectal neoplasms [4]. This, coupled with advances in the scope and efficacey of modern neoadjuvant therapies, has resulted in a significant increase in the proportion of rectal cancers potentially ameanable to local excision [5].

Current national comprehensive cancer network (NCCN) guidelines recommend transanal excision of low-risk T1N0 rectal tumours [1]. There are two predominant rectum sparing approaches to transanal excision: transanal endoscopic microsurgery (TEM) and transanal minimally invasive surgery (TAMIS). TEM was first described by Gerhard Buess in 1984, whilst TAMIS was pioneered in 2010 and allows the use of familiar laparoscopic cameras and instruments used in collaboration with a flexible disposable transanal access platform [6, 7]. Whilst a number of recent publications have explored the incidence of LARS following TEM, the incidence of LARS post-TAMIS has not previously been examined in the literature [8, 9]. The objectives of this study were to perform a prospective study evaluating rates of LARS post-TAMIS for the management of benign and malignant rectal neoplasms and explore patient, operative and tumour characteristics potentially predictive of LARS in this context.

## Materials and methods

### Data collection

This prospective pilot study included a consecutive series of patients undergoing TAMIS by a single surgeon at a single institution between January 2021 and February 2022. Indications for TAMIS included benign neoplasias not ameanable to endoscopic excision, or AJCC stage I (T1N0M0 or T2N0M0) rectal cancers recommended for TAMIS following multidisciplinary team discussion. The following patient, operative and tumour characteristics were collected from patient records: age, gender, body mass index (BMI), smoking status, American Society of Anaesthiologists(ASA) physical status score, Charlson co-morbidity index (CCI), length of inpatient stay (days), tumour histopathology findings, distance to the anal verge (based on pre-operative MRI or endoscopy report), operative approach (hybrid/pure), resection depth (submucosal/full thickness), post-operative complications and tumour location (anterior/posterior/right/left).

The LARS score is an internationally validated tool used to evaluate bowel dysfunction [10]. It consists of 5 questions that demonstrate high convergence between LARS and quality of life [11]. The score ranges from 0 to 42 points, with classification of patients into No LARS (0–20 points), Minor LARS (21–29 points), and Major LARS (30–42 points). LARS scores were recorded for each patient: pre-operatively (on the morning of surgery) at 1 month post-surgery and at 6 months post-surgery. The pre-operative LARS score was recorded in person on each occasion, whilst post-surgery LARS scores were recorded via telephone questionnaire. This research was approved by the Beaumont hospital Research and Ethics Committee, and all patients included provided written consent in advance of inclusion.

### Surgical technique

All procedures were performed by the same primary surgeon (JB), using the GelPOINT Path Transanal Access Platform (Applied Medical, Inc., Rancho Santa Margarita, CA). In some instances (hybrid approach), a Lone Star retractor (CooperSurgical Inc. Trumbull, CT) was also used for juxta-anal lesions. Pnuemorectum was maintained with CO_2_ insufflation with pressure set to 15 mmHg. A high-definition 10-mm camera lens was used in combination with standard laparoscopic graspers and a monopolar cautery device where feasible defect closure was performed using a V-Loc suture (Covidien-Medtronic, Minneapolis, MN).

### Statistical analysis

Patient, operative and tumour characteristics were compared between patients with No LARS or Minor/Major LARS at 6 months post-surgery. Statistical significance was determined using Mann–Whitney U test for continuous variables and Fishers exact test for categorical variables. Differences were considered significant if *p* < 0.05. Box and whisker plots were constructed to demonstrate interval change in LARS scores across the cohort; statistically significant changes in interval scores were determined using paired sample *t* test. All statistical analyses were performed using STATA 16.1 (StataCorp, College Station, TX).

## Results

A total of 20 patients were included in this prospective observational study. Fifty-five percent (*n* = 11) of patients were male, and mean age was 63 ± 12 years. Thirty percent (*n* = 6) of patients underwent TAMIS for management of an early rectal cancer, none of which received neoadjuvant chemoradiotherapy. All patients had an R0 resection. One patient had a post-operative bleed on day 3, which stopped spontaneously. No other peri- or post-operative complication was encountered. Defects were closed in all cases of full thickness excision, and there was no inadvertent peritoneal entry. Further patient, tumour and operative characteristics are outlined in Table 1.

**Table 1:** Patient, lesion, and operative characteristics

	All (*n* = 20)	No lars (*n* = 15)	Minor/major lars (*n* = 5)	*p* value
Age mean ± SD	63 ± 12	64 ± 12	61 ± 14	0.63
Male Sex *n*	11	9	2	0.62
BMI mean ± SD	29 ± 6	30 ± 7	28 ± 2	0.86
Smoker* n*	3	2	1	0.55
ASA* n*				0.65
I	3	3	0	
II	8	5	3	
III	9	7	2	
CCI *n*				0.45
0–3	10	7	3	
4–6	8	7	1	
> 6	2	1	1	
LOS (Days) *n*				**0.05**
0	18	15	3	
1	2	0	2	
Pathology				0.13
Adenoma* n*	14	12	2	
Villous	2	2	0	
Tubulovillous	12	10	2	
Adenocarcinoma* n*	6	3	3	
pT1	4	2	2	
pT2	2	1	1	
Positive margin (R1)* n*	0	0	0	1.00
Distance to anal verge (cm) mean ± SD	5.7 ± 3.2	6.4 ± 3.2	3.6 ± 2.1	**0.05**
Size (mm) mean ± SD	46 ± 20.5	45 ± 23.4	49.4 ± 7.4	0.23
Operative setup* n*				0.11
Pure	12	11	1	
Hybrid	8	4	4	
Resection depth* n*				1.00
Full thickness	16	12	4	
Submucosal	4	3	1	
Location *n*				1.00
Anterior	3	2	1	
Posterior	5	4	1	
Right	6	5	1	
Left	6	4	2	

Data are presented as *n* for categorical variables and mean ± SD for continuous variables. Patients were categorised into No/Minor/Major LARS according to their LARS score recorded at 6 months post-surgery

*BMI* body mass index, *ASA* American Society of Anaesthesiologists Grade, *CCI* Charlson comorbidity index, *LOS* length of stay

### LARS scores

**Fig. 1:**
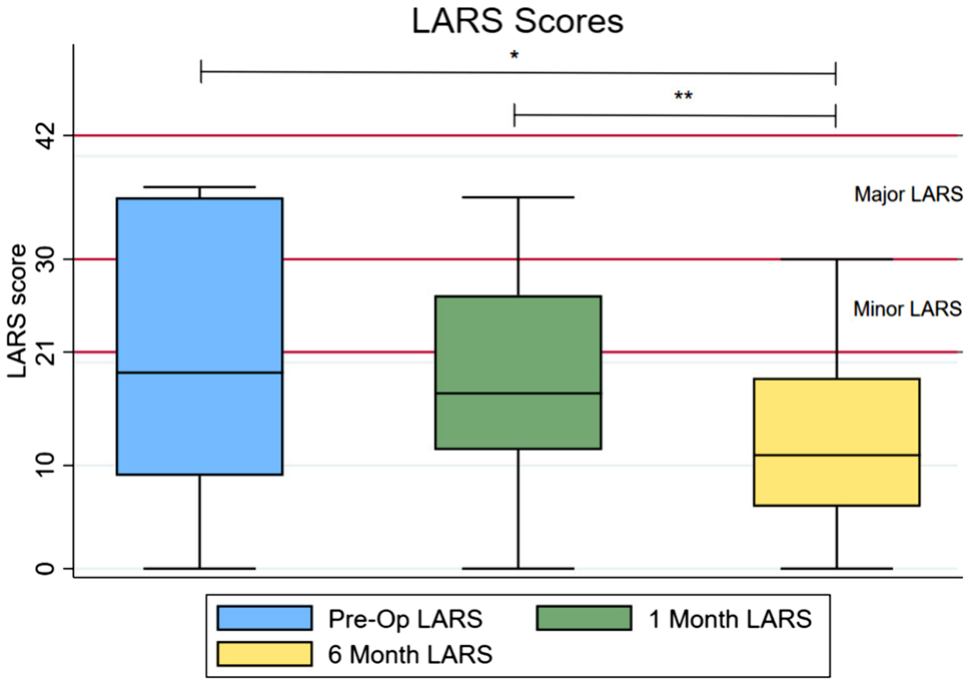
Box plots demonstrating pre-operative, 1-month and 6-month LARS scores. *p = 0.02, **p = 0.01

The mean interval LARS scores were 20.3 ± 12.9 pre-operatively, 18.2 ± 10.6 at 1 month post-surgery, and 12.6 ± 9.7 at 6 months after surgery. A statistically significant interval change was observed between pre-operative and 6-month post-operative LARS scores (*p* = 0.02) and also between 1-month and 6-month post-operative scores (*p* = 0.01) (see Fig. 1.)

Thirty percent (*n* = 6) of patients were found to have major LARS pre-operatively, and this reduced to 25% (*n* = 5) at 1 month and only 5% (*n* = 1) at 6 months. Meanwhile, 50% (*n* = 10) had No LARS pre-op, and this increased marginally to 55% (*n* = 11) at 1 month and significantly to 75% (*n* = 15) at 6 months. Twenty percent (*n* = 4) of patients were found to have Minor LARS pre-operatively, at 1 month post-op and 6 months after surgery. Of those patients with Major LARS pre-operatively, 3/6 (50%) were categorised as No LARS at 6 months, 2/6 (33%) had symptoms of Minor LARS at 6 months, whilst 1 patient (16.67%) continued to have symptoms of Major LARS at 6 months. Increased length of inpatient stay (*p* = 0.05) and reduced distance from the anal verge (*p* = 0.05) were two characteristics associated with LARS at 6 months (Table 1).

## Discussion

The incidence of Major LARS across our cohort was 5% at 6-month follow-up post-TAMIS. This compares favourably to functional outcomes post anterior resection, where Major LARS is experienced in up to 45% of post-operative patients [2, 3].

The precise pathophysiological mechanisms behind LARS remain unclear; however, it is thought to be multifactorial, likely occurring as a consequence of anal sphincter damage combined with alterations in neorectal configuration and motility resulting in disturbance of continence and evacuatory function [2]. The existing data pertaining to functional outcomes post-TEM, where there is no anastamosis or neorectal construction, report contrasting post-operative functional outcomes [8, 9]. Van Heinsbergen et al. found 29% of patients exhibited symptoms of ‘Major LARS’ post-TEM for management of Stage 1 rectal cancer, whilst Rizzo et al. report an incidence of ‘Major LARS’ in only 6.4% of patients on long-term follow-up post-TEM [8, 9]. A critical finding from both studies was the impact of neoadjuvant treatment on functional outcomes [8, 9]. No patients in our cohort received neoadjuvant therapy, and this may well help explain the significanct difference in reported post-operative LARS scores amongst our cohort and those patients included in the publication by van Heinsbergen et al., where 1 in 3 patients were in receipt of neoadjuvant treatment [9]. From a comparitive perspective, one of the most significant differences between TEM and TAMIS is the nature of the transanal access platform. TEM resectoscopes are metallic, rigid platforms fixed to the operative table by a mounting arm, whilst TAMIS adopts a shorter flexible disposable platform, which is not fixed and is simply held in place by ‘hooking’ into the anorectal ring [6]. Though the data from this pilot study are insufficient to suggest superiority of one minimally invasive modality over another, the authors hypothesise the less robust nature of the TAMIS platform may impact post-operative functional outcomes. Based on our findings, we propose a matched case control study inclusive of pre-operative LARS scores, in which patients who were or were not in receipt of neoadjuvant therapy would be grouped seperately for analaysis. This we feel would be beneficial in determining whether TAMIS may truly infer a benefit over TEM with regard to incidence of post-operative LARS.

A further striking finding from this study was the significant reduction observed between the pre-operative and 6-month post-operative LARS scores. Interestingly, 30% of patients (*n* = 6) described symptoms of Major LARS in advance of any surgical intervention. To our knowledge, pre-operative LARS scores have never previously been conducted in this context. On review of the characteristics of this specific cohort, the mean lesion diameter was 47.6 mm and mean distance to sphincter complex was 4.2 cm. Further analysis of the symptom profile of those patients with pre-operative Major LARS found that all 6 patients scored maximum points on questions 4 and 5 of the questionnaire (Q4. ‘*Do you ever have to open your bowels again within 1 h of the last opening?*’, Q.5 ‘*Do you ever have such a strong urge to open your bowels that you have to rush to the toilet?*’). Five out of 6 patients reported major improvements in both categories after the 6-month interval. The implication here is that large lesions in close proximity to the sphincter complex may cause a degree of sphincter irritation, presenting a symptom profile similar to LARS.

The limitations of this study include the small, heterogenous nature of the cohort. Follow-up time was also somewhat limited; however, available evidence suggests that though a partial improvement in symptoms may occur up to 12 months, many patients will have permanent symptoms beyond 6 months [12].

We noted a high prevalence of LARS across our cohort pre-operatively, and this had improved significantly at 6 month follow up after TAMIS. This is the first study of its kind to explore prevalence of LARS post-TAMIS, and it is the hope of the authors that this pilot study should prompt further investigations exploring potential functional benefits of TAMIS over alternative interventions.
